# First report of barley root-knot nematode, *Meloidogyne naasi* from turfgrass in Idaho, with multigene molecular characterization

**DOI:** 10.2478/jofnem-2023-0051

**Published:** 2023-11-20

**Authors:** Andrea M. Skantar, Zafar A. Handoo, Mihail R. Kantor, Maria N. Hult

**Affiliations:** Mycology and Nematology Genetic Diversity and Biology Laboratory, USDA, ARS, Northeast Area, Beltsville, MD 20705, USA; Department of Plant Pathology and Environmental Microbiology, Pennsylvania State University, University Park, PA 16802, USA

**Keywords:** golf course, identification, molecular markers, morphology, taxonomy

## Abstract

Barley root-knot nematode, *Meloidogyne naasi* Franklin, 1965, is one of the most important pest nematodes infecting monocots (Franklin, 1965). Two-inch core soil samples collected from a golf course in Ada County, Idaho were submitted for identification in November of 2019. A high number of *Meloidogyne* sp. juveniles were recovered from both soil samples using sieving and decantation followed by the sugar centrifugal flotation method. They were examined by light microscopy, morphometric measurements, and multiple molecular markers, including the ribosomal 28S D2–D3 and intergenic spacer 2 (IGS-2) regions, mitochondrial markers cytochrome oxidase I (COI) and the interval from COII to 16S, and the protein-coding gene Hsp90. Morphometrics as well as BlastN comparisons with other root-knot nematode sequences from GenBank were consistent with identification as *M. naasi*. Phylogenetic trees inferred from 28S, IGS-2, COI, or Hsp90 alignments each separated the Idaho population into a strongly supported clade with other populations of *M. naasi*, while the COII-16S interval could not resolve *M. naasi* from *M. minor*. This report represents the first morphological and molecular characterization of *Meloidogyne naasi* from turfgrass in Idaho.

Turfgrasses are widely used as groundcovers in a variety of sporting, residential, and recreational contexts, and maintaining high quality and aesthetics is an essential feature of turfgrass management. Control of newly emerging diseases and nematodes presents an ongoing challenge to the health, quality, production, and maintenance of turfgrass. The barley root-knot nematode, *Meloidogyne naasi* Franklin, 1965, was originally described in field crops (cereals, grasses, and sugarbeet, *Beta vulgaris* L.) in England and Wales (Franklin, 1965). This species is one of the most important root-knot nematodes (RKN) affecting monocots, and is known to cause reduced growth and vigor in turfgrasses (Bélair et. al., 2006). This species has a wide distribution, having been reported in the United States in several states, as well as in Canada, South America and Europe (Zeng et al., 2012). A report from 2012, in which authors surveyed 238 golf courses from 10 states across the western United States, found RKN in 60% of the samples (McClure et. al. 2012). Molecular analysis of specimens from 110 golf courses revealed that the most prevalent root-knot nematode species was *M. naasi,* which was found in 58 samples (McClure et al., 2012).

A similar study by Ye et al. (2015) analyzed data from 51 RKN populations collected from turfgrasses within the eastern United States. In contrast with the western U.S. golf courses surveyed, the most prevalent RKN species on the East Coast were *M. marylandi* and *M. graminis* (McClure et al., 2012; Ye et al., 2015). *Meloidogyne naasi* was also identified, but was not present in as many samples as *M. marylandi* and *M. graminis* (Ye et al., 2015). More recently, *Meloidogyne naasi* was found on a football field in Porto, Portugal (Vieira dos Santos et al., 2020).

Molecular markers continue to be a useful tool for distinguishing *Meloidogyne* species, particularly ribosomal large subunit (LSU) D2–D3 segments of the 28S rDNA (Skantar et al., 2021; Trinh et al., 2022; Zeng et al., 2022) and the mitochondrial DNA interval that spans the region between genes encoding cytochrome oxidase subunit II (COII) and 16S rRNA (Powers and Harris, 1993; Subbotin, 2021; Subbotin and Burbridge, 2021). IGS-2 has been sequenced from several RKN species, but not from *M. naasi.* Mitochondrial cytochrome oxidase 1 (COI) has gained popularity as a molecular barcode for many nematodes and is useful for differentiating some RKN species (*M. fallax, M. chitwoodi, M. minor, M. naasi,* and *M. hapla*) (Alvarez-Ortega, 2019; Powers et al., 2018; Skantar et al., 2021), as is the nuclear protein coding gene heat shock protein 90 (Hsp90) (Nischwitz et al., 2013).

The Banbury Golf Course in Bandon, Oregon reported a poorly performing section that had been planted entirely from seed, including a majority percentage of *Poa annua* (via overseeding the original bentgrass/fescue seed mix) (Fig. 1). Preliminary analysis by Rutgers University identified *Anguina pacificae* in these samples, but subsequent examination by diagnosticians at Oregon State University and our laboratory failed to repeat that finding. From the two soil samples received for identification, only juveniles were recovered. No mature females or males were found. The morphometric details of the juveniles were recorded and compared to the original description of *M. naasi* (Franklin, 1965) and others that followed (Karssen et al., 2002; Zhao et al., 2017, Suresh et al., 2017 and Santos et al., 2020). Sequences from the large subunit ribosomal DNA (28S), intergenic region (IGS-2), the mitochondrial DNA interval between cytochrome oxidase II (COII) and the 16S gene, partial mitochondrial cytochrome oxidase I (COI), and nuclear heat shock protein (Hsp90) gene were obtained and compared to the existing information in GenBank. Based upon the unambiguous similarity of these DNA markers with those previously reported for the species (McClure et al., 2012; Nischwitz et al., 2013) and the morphological data, we identify this isolate as *Meloidogyne naasi*. Prior evidence of *M. naasi* in Idaho was reported by Powers et al. (2018), who recovered a COI sequence from RKN on potato. This report represents the first morphological and molecular description of *Meloidogyne naasi* from Idaho turfgrass.

**Figure 1:**
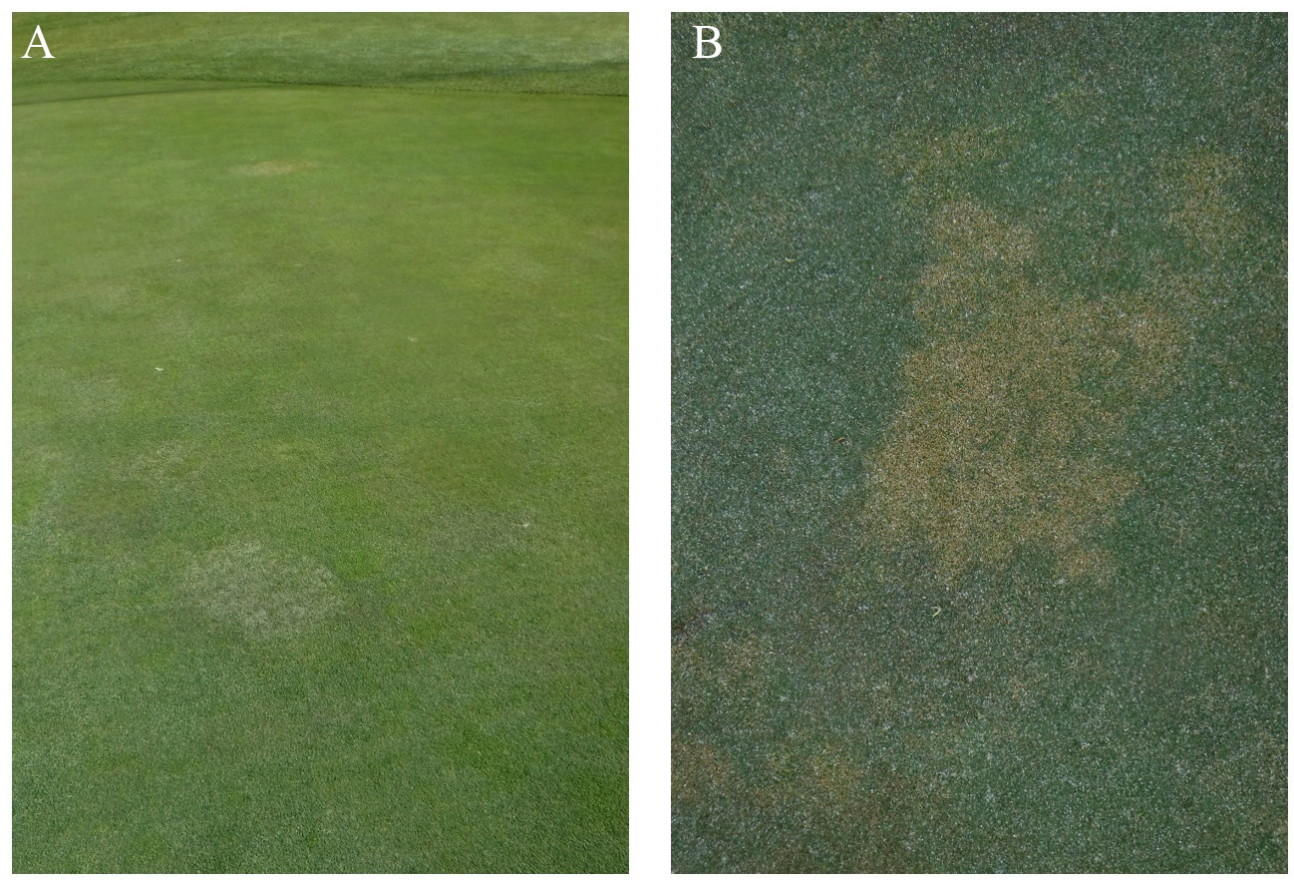
Damage on golf course turfgrass in Banbury, Idaho. A. Golf course green in decline, showing patchy surface. B. Close-up of damage to mixed turfgrass, associated with *M. naasi*. Photo credits: Darryl Glinski.

## Materials and Methods

Two soil samples were sent to the USDA Mycology and Nematology Genetic Diversity and Biology Laboratory (Beltsville, MD) in November of 2019. The origin of the soil samples was Banbury Golf Course in Ada County, Idaho. Nematodes were recovered from soil using nested 20, 60, and 500 μm sieves, with juveniles (J2s) recovered from the 500 μm sieves using the sieving decanting method, followed by sugar centrifugal flotation (Jenkins, 1964). Nematodes were fixed in 3% formaldehyde and processed to glycerin by the formalin glycerin method (Golden, 1990; Hooper, 1970). Females recovered from the soil samples were fixed in 3% formaldehyde solution. Photomicrographs of the specimens were made with a Nikon Eclipse Ni compound microscope, using a Nikon DS-Ri2 camera (Nikon USA, Melville, NY). Measurements were made with an ocular micrometer on a Leica WILD MPS48 Leitz DMRB compound microscope (Leica, Wetzlar, Germany). All measurements are in micrometers unless otherwise stated.

The molecular identification was performed using DNA extracted from three separate juveniles (J2) as templates in PCR reactions. Individual J2 were mechanically disrupted in 20 μl of extraction buffer (125 mM KCl, 25 mM Tris-Cl, pH 8.3, 3.75 mM MgCl_2_, 2.5 mM DTT, 1.125% Tween 20), then stored in PCR tubes at −80 °C until needed. To prepare DNA extracts, frozen nematodes were thawed, 1 μl proteinase K (from 2 mg/ml stock solution) was added, and tubes were incubated at 60 °C for 60 min, followed by 95 °C for 15 min to deactivate the proteinase K and centrifuged briefly prior to use in PCR. All PCR reactions contained 0.75 μl of each primer and 0.125 μl of Dream Taq (0.625 Units) per 25 μl reaction. The 28S reaction included 2 μl of DNA template; the rest contained 3 μl. Ribosomal PCR products were amplified from the 28S D2–D3 expansion segment as described by De Ley et al. (2005) with primers D2A (5′- 1334–1340) and D3B (5′-TCGGAAGGAACCAGCTACTA-3′), and from the intergenic spacer (IGS-2) according to Blok et al. (2002) with primers 5S (5′-TTAACTTGCCAGATCGGACG-3′) and 18S (5′-TCTAATGAGCCGTACGC-3′). Mitochondrial DNA products were amplified from the variable-marker region between the mitochondrial COII and the large (16S) rRNA gene, as described by Powers and Harris (1993), with primers 1RNAF (5′-TACCTTTGACCAATCACGCT-3′) and COIIR (5′-GGTCAATGTTCAGAAATTTGTGG-3′), and partial COI according to Derycke et al. (2010), with primers JB3 (5′-TTTTTTGGGCATCCTGAGGTTTAT-3′) and JB5 (5′-AGCACCTAAACTTAAAAC ATAATGAAAATG-3′). Hsp90 sequences were amplified according to Nischwitz et al. (2013) with primers RKN-d1F (5′- GCYGATCTTGTYAACAACCYTGGAAC-3′) and RKN-5R (5′-TCGAACATGTCAAAAGGAGC-3′). PCR products were cleaned with the Monarch DNA Gel Extraction Kit (NEB, Ipswitch, MA), and Hsp90 products were cloned using the Strataclone PCR Cloning Kit (Agilent, Santa Clara, CA), prepared with the Monarch Plasmid Miniprep Kit (NEB), and sequenced with vector M13F and M13R primers.

Other PCR amplicons were sequenced directly with their corresponding PCR primers, except the 28S which was sequenced with the primers D3A and D3B. All PCR and cloned products were sequenced by Genewiz, Inc. (South Plainfield, NJ, USA). GenBank accession numbers were assigned as follows: 28S rDNA (MT406252); IGS-2 rDNA (OQ72994 to OQ72997); mitochondrial COII to 16S DNA (MT408951 to MT408953); COI (MT408951 to MT408953); and Hsp90 (MT408947 to MT4089450). Newly obtained sequences were compared with publicly available sequences in GenBank using BlastN. Multiple sequence alignments of each marker were constructed with ClustalW or MAFFT within Geneious, with outgroups chosen based on previous studies. Phylogenetic trees were constructed by Bayesian Inference (BI) under the GTR + I + G model implemented within the MrBayes plugin in Geneious. BI analysis for each gene included a random starting tree and was run with four chains for 2 × 10^6^ generations. Two runs were performed for each analysis. Trees were sampled every 1000^th^ generation, with 25% of results discarded as burn-in. Remaining samples were used to generate a 50% majority-rule consensus tree. Posterior probabilities (PP) are shown on appropriate clades.

## Results and Discussion

### Morphological Analysis

**Figure 2:**
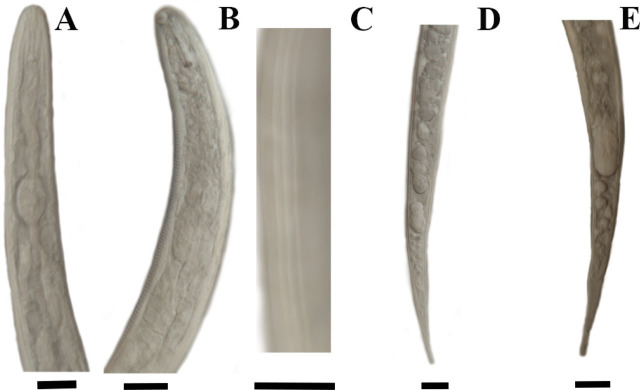
Photomicrographs of *Meloidogyne naasi* second-stage juveniles. A,B, heads; C, lateral lines; D,E, tails; the scale bar =10 μm.

Morphological measurements of *M. naasi* juveniles (n = 30) included body length, maximum body width, stylet length, body width at anus, tail length, tail terminus length, and a and c values (Table 1). Four lines were noted in the lateral field. All measurements were consistent with values previously established for *M. naasi* (Franklin, 1965; Karssen et al., 2002; Zhao et al., 2017, Suresh et al., 2017 and Santos et al., 2020). Images from second-stage juveniles are shown in Fig. 2.

**Table 1:** Morphometrics of infective second-stage juveniles of *Meloidogyne naasi*. Values expressed in the form mean +/− standard deviation (range).

**Characters**	**Idaho Second-stage juveniles (n = 30)**	***M. naasi* (Franklin, 1965) (n = 25)**	***M. naasi* (Karssen et al., 2002) (n = 4)**	***M. naasi* (Zhao et al., 2017) (n = 17)**
Linear (μm)				
Body length	427.9 ±16.73 (390 – 460)	435 (418 – 465)	421 ± 8.1 (410 – 429)	429 ± 16.1 (397 – 467)
Maximum body width	16.2 (15.0 – 17.5)^*^	15 ± 0.95 (14 – 17.5)	14.1 ± 0.6 (13.3 – 14.5)	15.9 ± 0.7 (14.4 – 17.3)
Stylet length	12.09 ± 0.56 (11.0 – 13.0)	14 (13 – 15)	13.3 ± 0.5 (12.6 – 13.9)	11.7 ± 0.6 (10.8 –1 2.4)
Body width at anus	10.3 ± 0.9 (9.0 – 12.0)^*^	11 (9 –13)	-	-
Tail length	70.3 ± 5.04 (62.0 – 80.0)	70 (52 – 78)	66.0 ± 3.9 (61.0 –70.0)	68.2 ± 8.0 (55–78)
Hyaline tail terminus length	21.4 ± 2.16 (17.0 – 26.0)	n.d.	17.9 ± 1.8 (15.8 – 19.6)	24.7 ± 2.6 (19.6 – 29.8)
Lines in lateral field	4	4	-	-
Ratios				
a = body length/greatest body diameter	35.61 ± 2.21 (32.24 – 40.91)	28 (25 – 32)	30 ± 1.5 (28.3 – 31.8)	27.1 ± 1.9 (24.2 – 30.7)
c = body length/tail length	6.11 ± 0.43 (4.94 – 6.79)	6.2 (n.d.)	6.4 ± 0.3 (6.0 – 6.7)	6.4 ± 0.8 (5.6 – 8.0)

* Values were calculated based on 10 specimens.

### Molecular Analysis

Amplification of the IGS-2 ribosomal DNA region and the D2–D3 expansion segment of region 28S yielded sequences of 1334 to 1340 bp and 775 bp, respectively. Amplification of the mitochondrial interval between COII and the large (16S) rRNA gene yielded sequences of 416 to 441 bp, and from the COI gene, 752 to 796 bp. Hsp90 PCR generated sequences ranging from 1031 to 1043 bp.

**Figure 3:**
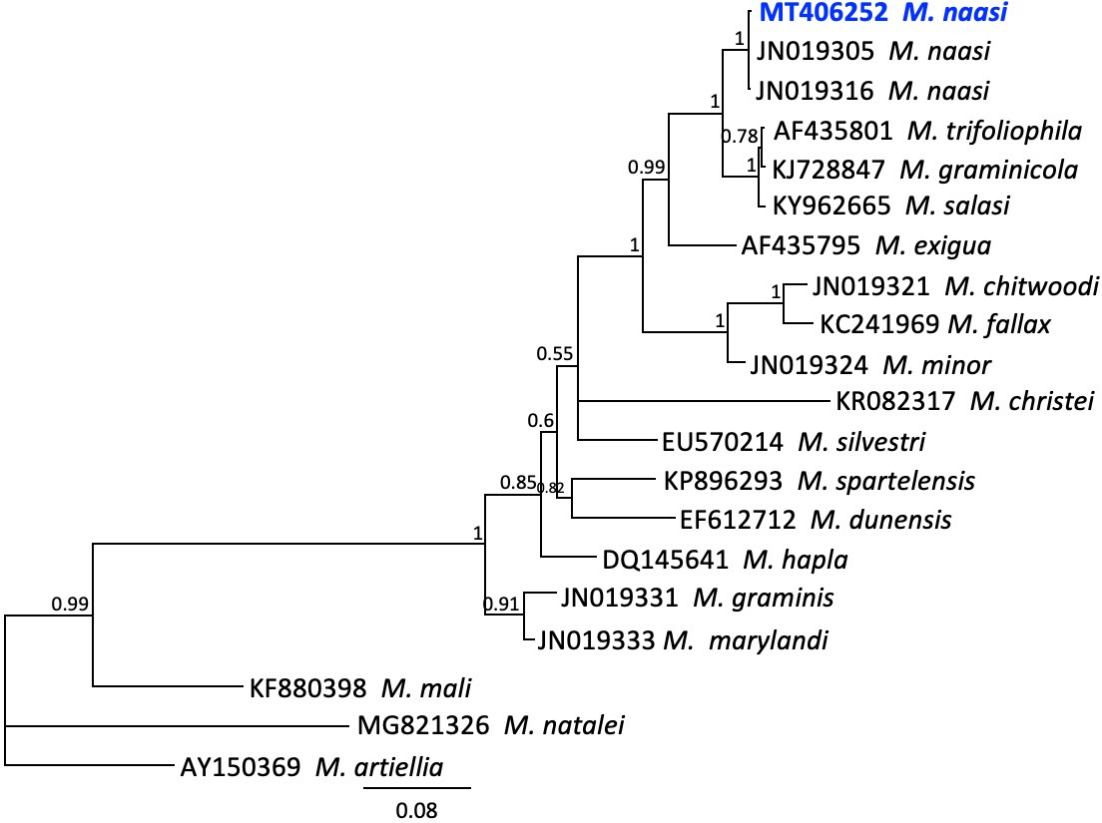
Phylogenetic relationships of Idaho population of *Meloidogyne naasi* and other selected root-knot nematodes, as inferred from a 735-bp alignment of 28S rDNA sequences, with *M. artiellia* as the outgroup. A 50% majority rule consensus tree obtained from Bayesian analysis was generated using the GTR + I + G model of nucleotide substitution. Branch support (PP) values above 0.5 are shown on appropriate branches. New sequences are highlighted in blue bold font.

Amplification of the D2–D3 expansion region of 28S rDNA yielded identical sequences from three individual J2. Similarity matches from BlastN against the GenBank database included other *M. naasi* sequences at 100%, with full coverage. These matches included accessions KP901068 and KP901069 representing populations obtained from bentgrass in NC, and several others from the U.S. (McClure *et al.,* 2012) and Portugal (Vieira dos Santos *et al.*, 2020), with > 99% similarity (2 to 9 bp differences). Bayesian phylogenetic trees constructed from multiple sequence alignments of *Meloidogyne* spp. placed the Idaho population in a clade with other *M. naasi* sequences at PP = 1.00 support Fig. 3. This clade was in a sister group with *M. trifoliophila, M. graminicola* and *M. salasi* with maximal support.

Amplification of the IGS-2 rDNA from three J2 gave two identical sequences and a third that was 99.6% identical (7 bp differences). These IGS-2 sequences from *M. naasi* are the first to be recovered from this species, and no others were available in GenBank for comparison. Four additional populations identified as *M. naasi* in a previous study from golf course turfgrass were also subjected to PCR of IGS-2 (McClure et al., 2012). These sequences ranged from 99.3 to 100% identical (0 to 9 bp differences) to the Idaho population. The other highest similarity matches included *M. oryzae* (LS974439), with 73% identity.

**Figure 4:**
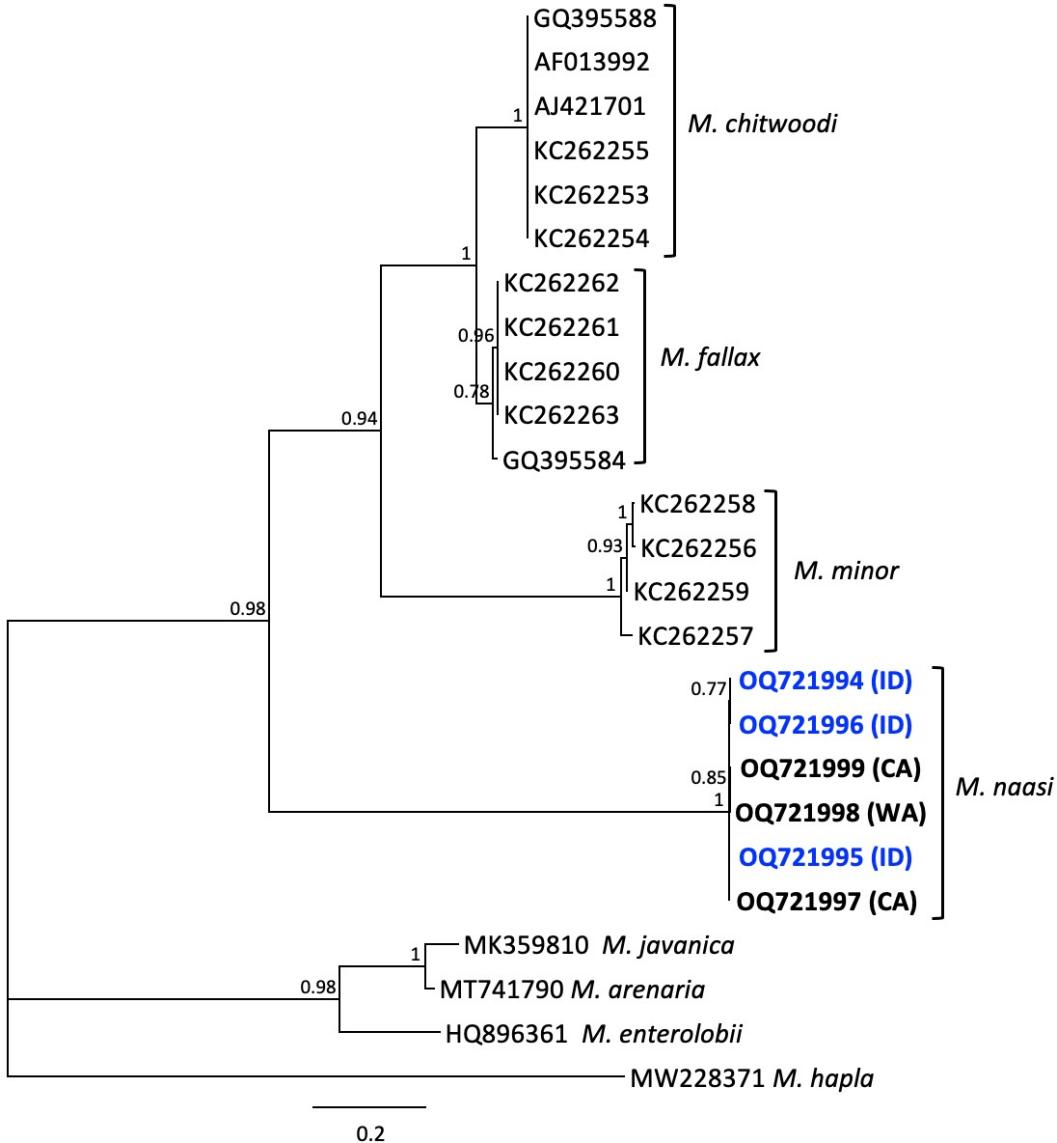
Phylogenetic relationships of Idaho population of *Meloidogyne naasi* and other selected root-knot nematodes, as inferred from a 1764-bp alignment of IGS-2 rDNA sequences, with *M. hapla* as the outgroup. A 50% majority rule consensus tree obtained from Bayesian analysis was generated using the GTR + I + G model of nucleotide substitution. Branch support (PP) values above 0.5 are shown on appropriate branches. New sequences are highlighted in blue bold font.

The phylogenetic tree (Fig. 4) inferred from alignment of newly obtained *M. naasi* IGS-2 and other RKN sequences placed the Idaho population in a clade along with two *M. naasi* isolates from California and one from Washington with maximum support (PP = 1.0). The *M. naasi* sequences were grouped near *M. minor* and a clade containing *M. fallax* and *M. chitwoodi.* Other IGS-2 sequences from species included in the 28S tree were not available for further comparison of their topologies.

**Figure 5:**
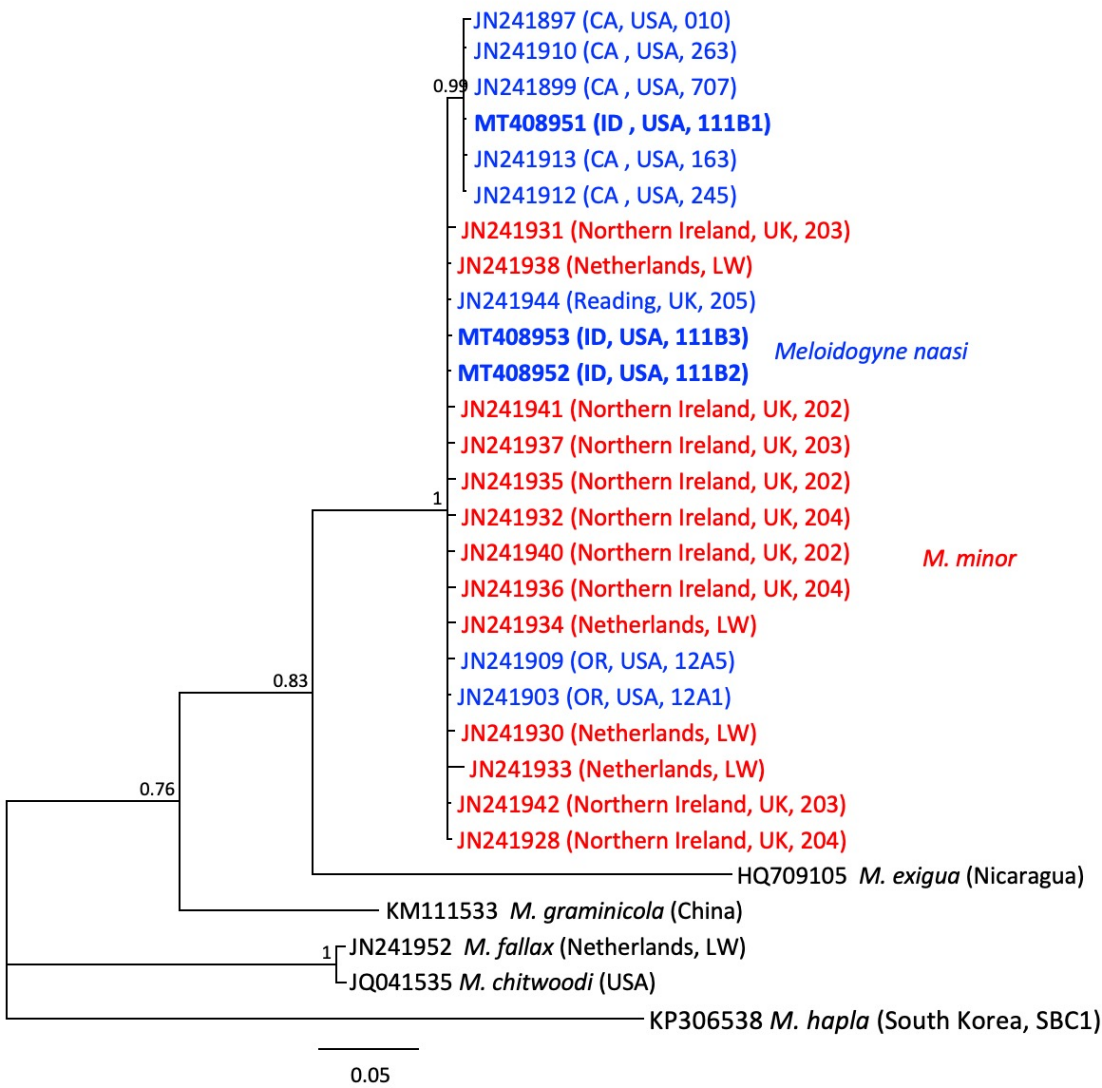
Phylogenetic relationships of Idaho population of *Meloidogyne naasi* and other selected root-knot nematodes, as inferred from a 1764-bp alignment of mitochondrial sequences including the interval from COII to 16S, with *M. hapla* as the outgroup. A 50% majority rule consensus tree obtained from Bayesian analysis was generated using the GTR + I + G model of nucleotide substitution. Branch support (PP) values above 0.5 are shown on appropriate branches. New sequences are highlighted in bold font; other *M. naasi* is in blue and *M. minor* in red font.

PCR of the mitochondrial interval COII-16S from three J2 gave two identical sequences, and a third that varied at 3 bp from the others (99.6 to 100% identity). BlastN yielded top hits to both *M. naasi* and *M. minor* sequences (between 0 and 7 bp differences, with 99.1 to 100% identity). The Bayesian tree constructed from a multiple sequence alignment of selected *Meloidogyne* populations (Fig. 5) showed that *M. naasi* and *M. minor* formed a polyphyletic clade, and that COII-16S could not reliably resolve these species.

In a previous study of RKN populations from golf course turfgrass (McClure et al., 2012), mtCOII-16S interval could not distinguish populations *M. fallax* and *M. chitwoodi,* with sequences from several geographically diverse populations differing at only a single deletion or insertion. Thus, this locus is not able to resolve some RKN species.

The presence of mixed *M. naasi* and *M. minor* populations on golf-course turfgrass has created challenges for molecular species discrimination, and care must be taken to ensure that specimens in a mixture are carefully separated before molecular analysis. *Meloidogyne minor* is a relatively new species first found on potato in the Netherlands in 2004, on sports fields in the United Kingdom (Karssen et al., 2004), and in Belgium, Chile, and New Zealand (Zhao et al., 2017). Unfortunately, mtCOII-16S sequences from the latter countries were unavailable, precluding a broader geographic comparison of diversity in this marker.

Amplification of mitochondrial COI gave two identical sequences from separate J2. BlastN comparison to GenBank resulted in matches to *M. naasi* sequences KU517170 (origin not given) and MH128479 (reported from potato in Idaho; Powers et al., 2018) at between 99.4 and 99.5% identities (2 to 3 bp differences). Additional *M. naasi* matches included longer COI sequences (763 bp) generated with different primers (Powers et al., 2018) that varied at 20 to 23 bp over a 397-bp region in common. Sequences from *M. naasi* (KM491208, KM49121 to KM49125) only overlapped at 105 bp with the fragments generated here, with those matching 100%.

**Figure 6:**
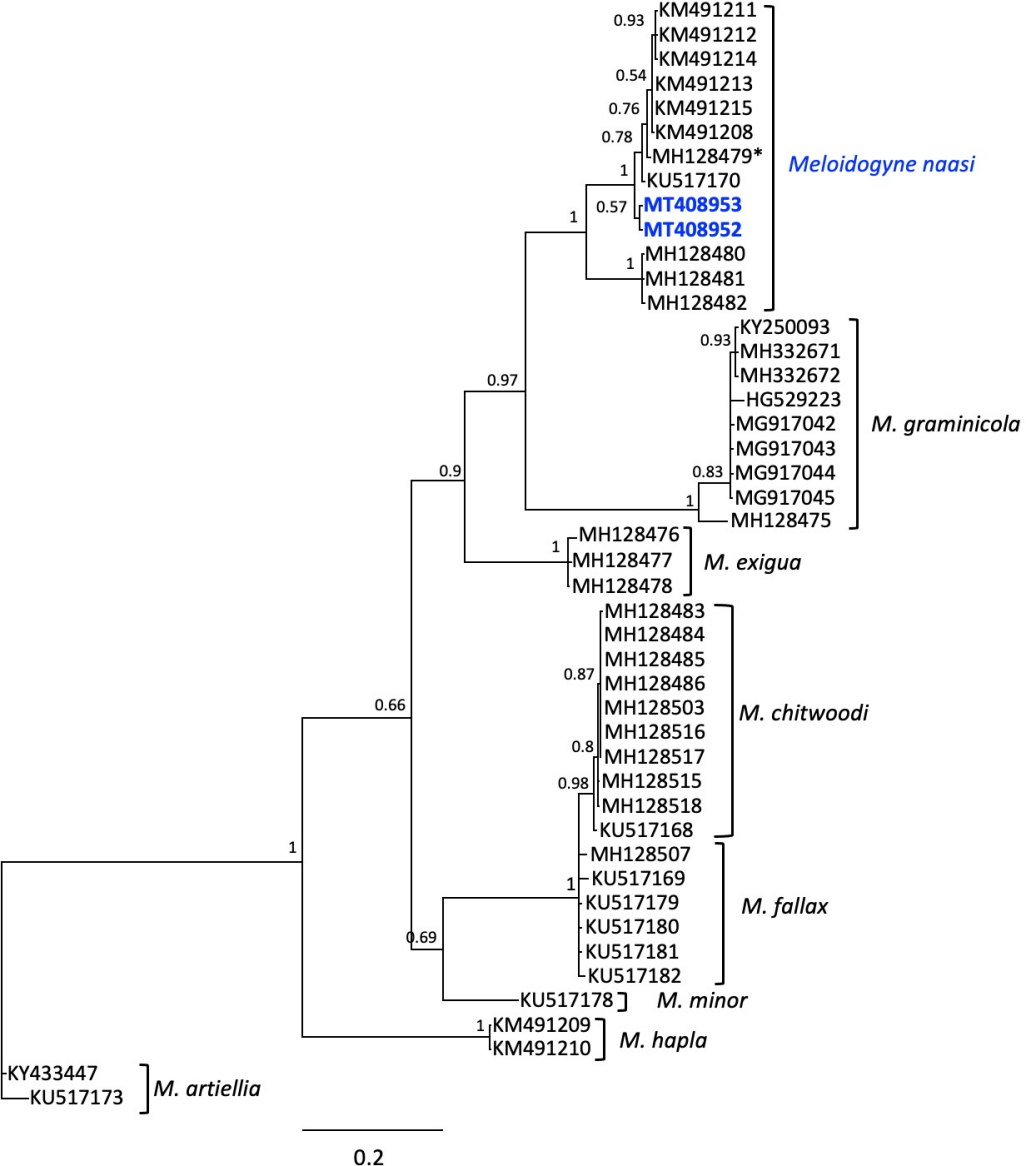
Phylogenetic relationships of Idaho population of *Meloidogyne naasi* and other selected root-knot nematodes, as inferred from a 763-bp alignment of mitochondrial COI sequences, with *M. artiellia* as the outgroup. A 50% majority rule consensus tree obtained from Bayesian analysis was generated using the GTR + I + G model of nucleotide substitution. Branch support (PP) values above 0.5 are shown on appropriate branches. New sequences are highlighted in blue bold font. The COI sequence from Powers et al. (2018) is marked by an asterisk.

In the Bayesian tree resulting from the multiple sequence alignment generated with selected RKN species (Fig. 6), all *M. naasi* sequences formed a clade with maximum PP = 1.00. Sequences from *M. chitwoodi* and *M. fallax* grouped together, separated by 5 bp, as shown in a prior study (Powers et al., 2018).

**Figure 7:**
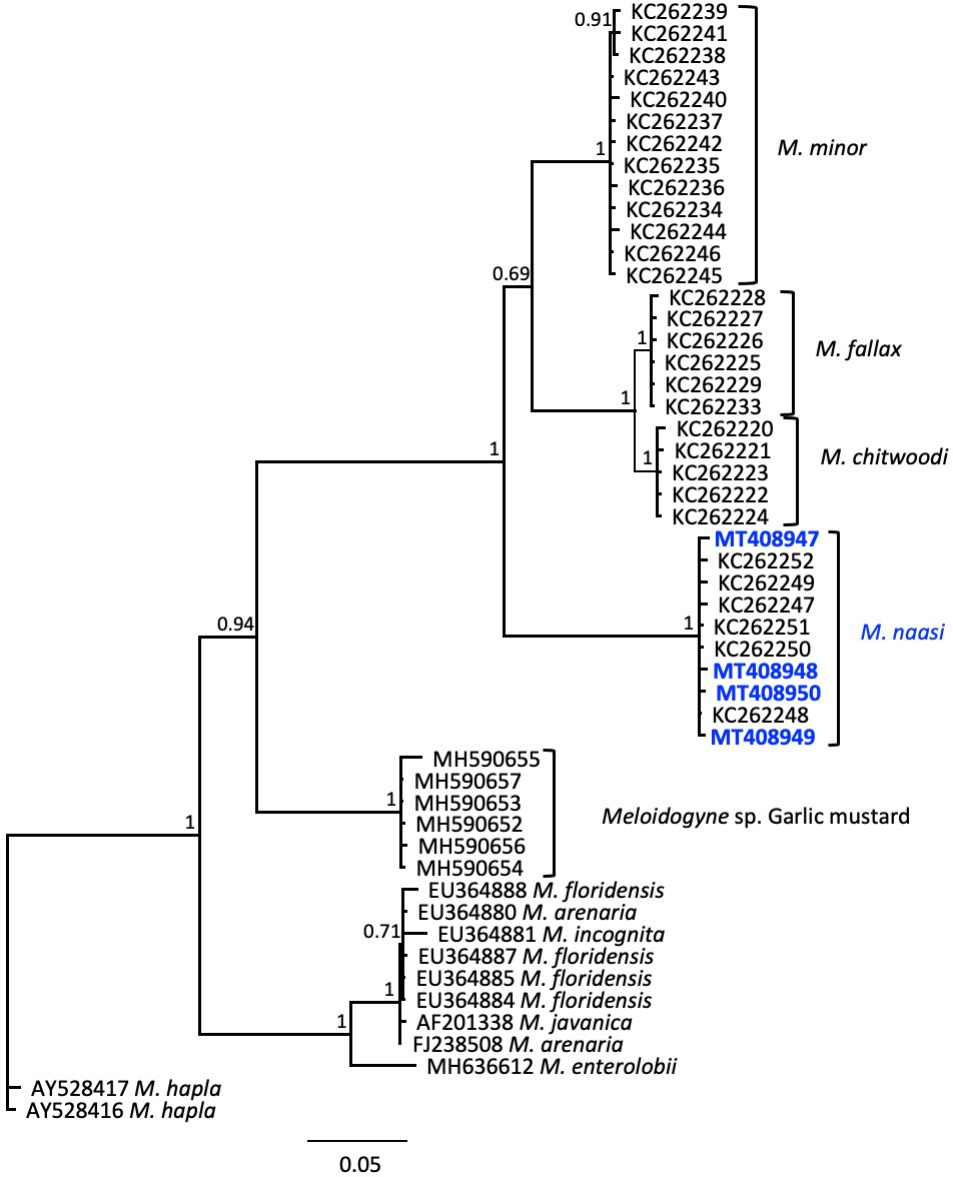
Phylogenetic relationships of Idaho population of *Meloidogyne naasi* and other selected root-knot nematodes, as inferred from a 1003-bp alignment of Hsp90 genomic sequences, with *M. hapla* as the outgroup. A 50% majority rule consensus tree obtained from Bayesian analysis was generated using the GTR + I + G model of nucleotide substitution. Branch support (PP) values above 0.5 are shown on appropriate branches. New sequences are highlighted in blue bold font.

Hsp90 amplification from three juveniles resulted in four cloned PCR products varying in length from 1031 to 1042 bp, with 98.8 to 99.4% identity (6 to 12 bp differences) among clones. BlastN comparison to sequences in GenBank indicated that the best matches to *M. naasi* were previously identified from golf courses in the western U.S. (KC262248 to KC262252) with 99.0 to 99.9% identity (1 to 9 bp differences). The next closest similarity was to *M. minor* (KC262245) and several others, at 85.47% similarity (145 bp differences). In the Bayesian tree inferred from alignment of selected RKN Hsp90 sequences (Fig. 7), *M. naasi* formed a maximally supported clade. Other distinct clades with maximum support values were obtained for *M. minor, M. fallax, M. chitwoodi,* and an unnamed RKN species isolated from garlic mustard. Tropical species *M. floridensis, M. incognita, M. arenaria,* and *M. javanica* could not be reliably separated by Hsp90, although *M. enterolobii* grouped separately from those species.

Based upon the unambiguous similarity of these DNA markers with those previously reported for the species (McClure et al., 2012, Nischwitz et al., 2013, Hodgetts et al., 2016; Powers et al., 2018) and the morphological data (Franklin, 1965; Karssen et al., 2002; Zhao et al., 2017, Suresh et al., 2017; Santos et al., 2020), we identify this isolate as *Meloidogyne naasi*. For each marker examined, the phylogenetic groupings of species were consistent, limited only by the availability of sequences from each species, with *M. naasi* appearing in Clade III, as defined previously in a 5-gene phylogeny by Alvarez-Ortega et al. (2019). This clade includes nine species from monocots and dicots that reproduce exclusively by meiotic parthenogenesis. Except for *M. salasi*, found only in South America, and *M. kralli*, found only in Europe, the rest of the species in Clade III (*M. trifoliophila, M. graminicola, M. naasi, M. exigua, M. chitwoodi, M. fallax,* and *M. minor*) are found on multiple continents.

The greens at Banbury Golf Course were originally planted as creeping bent grass (*Agrostis stolonifera ‘*Dominant Plus’). The course originated in 1999. The greens are currently a mixture of creeping bent grass and annual bluegrass (*Poa annua*). Nematode damage has been observed in the spring, summer, and fall. The patches often reappeared in identical or very similar locations. Most of the greens (20 total, including the practice putting green and the chipping green) have seen damage. A few show no signs of nematodes. Interestingly, the damage often impacts the bent grass and not the annual bluegrass (Darryl Glinski, personal communication).

This study represents the first report of *M. naasi* from turfgrass in Idaho, accompanied by micrographs, morphometrics, and multi-gene characterization. This adds to a previous study by Powers et al. (2018), who identified *M. naasi* from Idaho potato based on COI. The nematodes in the present work were isolated from soil samples taken from golf course greens, not from the roots themselves, although the turf was reported by the golf superintendent to be in decline. Interestingly, no other species of RKN were found in the samples. Mixtures of RKN species on golf course greens were previously reported by McClure et al. (2012), and indeed, some of the samples included in that study required careful microscopic examination of J2 to manually separate *M. naasi* from *M. minor*.

In the United Kingdom, mixed populations of *M. naasi* and *M. minor* are common. *Meloidogyne naasi* was the most prevalent RKN detected in a survey of 11 golf courses and eight football pitches in Belgium (Vandenbossche et al., 2011). In Washington State, these species were detected together on golf course greens in ratios of approximately 4:1. In a study of nematodes identified by diagnostic laboratories in the Pacific Northwest (Zasada et al., 2019), *M. naasi* was found in fewer than 1% of samples, making them much less common than *M. chitwoodi* (found in 71% of samples) and *M. hapla* (16%) or *M. hapla-*and-*M. chitwoodi* mixtures (12%). Contamination of shoes, clubs, and other equipment with infested soil may well contribute to further expansion of *M. naasi* in the region.

## Appendix

**Supplementary Table S1.** Root knot nematode isolates and GenBank numbers included in this study.

**Species**	**Origin**	**Sample ID**	**28S**	**IGS-2**	**mtCOI**	**COII-16S**	**Hsp90**
*M. arenaria*	Florida, USA	-					EU364880
	Maryland, USA	-					FJ238508
	Sri Lanka	-		MT741790			
*M. artiellia*	-	-			KY433447		
	-	-			KU517173		
	Italy	-	AY150369				
*M. chitwoodi*	-	NEMBAR210			KU517168		
	Elko Co., Nevada, USA	N7145			MH128483		
		N7147			MH128484		
		N7148			MH128485		
		N7149			MH128486		
	San Juan Co., NM	P221087			MH128518		
	San Luis Obispo Co, CA	186	JN019321				
	USA					JQ041535	
	Washington, USA	P21040			MH128515		
		P215031			MH128516		
		P215032			MH128517		
		13B					KC262220 (cl 1219)
							KC262221 (cl 1220)
							KC262222 (cl 1221)
							KC262223 (cl 1222)
							KC262224 (cl 1223)
*M. christei*	Florida, USA	-	KR082317				
*M. dunensis*	Spain		EF612712				
*M. enterolobii*	China			HQ896361			
	Florida, USA	03A1					MH636612 (cl. 842)
*M. exigua*	Brazil	-	AF435795				
	Nicaragua	-				HQ709105	
		N213			MH128476		
		N214			MH128477		
		N215			MH128478		
*M. fallax*	Netherlands^*^	LW				JN241952 (cl 2584)	KC262225 (cl 2735)
							KC262226 (cl 2736)
							KC262227 (cl 2734)
							KC262228 (cl 2737)
	CA, USA	853					KC262229
							KC262233
	San Francisco Co., CA	853	KC241969	KC262260–KC262263			
	Scotland, United Kingdom	P192084			MH128507		
	Switzerland	-		GQ395584			
	-	NEMBAR256			KU517169		
	United Kingdom	NEMBAR1145			KU517179		
		NEMBAR1174			KU517180		
		NEMBAR1180			KU517181		
		-			KU517182		
*M. floridensis*	Peach Co., Georgia, USA	Mf1					EU364884
		Mf3					EU364885
		Mf_GA2					EU364887
		Mf_GA3					EU364888
*M. graminicola*	China				KY250093		
					MG917042–MG917045		
		-				KM111533	
	Florida, USA	P169011			MH128475		
	Phillippines				HG529223		
	Taiwan		KJ728847				
	Vietnam				MH332671		
	Vietnam				MH332672		
*M. graminis*	San Diego, Co., CA	730	JN019331				
*M. hapla*	China			MW228371			
	Hawaii, USA						AY52841
	Maryland, USA^***^ (culture)						AY528417
							AF201338
	Netherlands	7J2			KM491210		
	Netherlands		DQ145641				
	Switzerland	6C1			KM491209		
	Qujing County, China			MK359810			
*M. javanica*	Japan		KF880398				
	Maricopa Co., Arizona, USA	001	JN019333				
*M. mali*	-	NEMBAR1327			KU517178		
*M. marylandi*	King Co., Washington, USA	438	JN019324				
*M. minor*	Netherlands^*^ LW	68D		KC262259		JN241933 (cl 2636)	KC262234 (cl. 2758)
						JN241934 (cl 2638)	KC262235 (cl. 2759)
						JN241938 (cl 2640)	KC262236 (cl. 2760)
						JN241930 (cl 2641)	KC262237 (cl. 2761)
	Northern Ireland, United Kingdom^*^	202		KC262256		JN241940 (cl 2618)	KC262242 (cl 2746)
						JN241935 (cl 2619)	
						JN241942 (cl 2620)	
						JN241941 (cl 2621)	
	Northern Ireland, United Kingdom^*^	203		KC262257		JN241931 (cl 2624)	
						JN241942 (cl 2625)	
						JN241937 (cl 2627)	
	Northern Ireland, United Kingdom^*^	204		KC262258		JN241928 (cl 2633)	KC262238 (cl 2748)
						JN241932 (cl 2634)	KC262239 (cl 2749)
						JN241936 (cl 2635)	KC262240 (cl 2751)
							KC262241 (cl 2750)
	-	NEMBAR070			KU517170		
*M. naasi*	Ada Co., Idaho, USA	111B1	MT406252	OQ721995		MT408951	MT408947 (cl 3870)
		111B2		OQ721994	MT408952	MT408952	MT408948 (cl 3872)
		111B3		OQ721996	MT408953	MT408953	MT408949 (cl 3874)
							MT408950 (cl 3875)
	Idaho, USA^**^	N326			MH128479		
	Jackson Co, Oregon, USA	410	JN019305				
	Lane Co., Oregon, USA	054				JN241903	
	Kitsap Co., Washington, USA	351	JN019316				
	King Co., Washington, USA	194		OQ721998			
	Linn Co., Oregon, USA	12A			KM491211		
					KM491212		
	Linn Co., Oregon, USA	010					
	Monterey Co., California, USA	707					
	Stanislaus Co., California, USA	263					
	San Luis Obispo, Co., California, USA	163					
	San Diego Co., CA						
	Linn Co., Oregon, USA	12A			KM491214		
	Monterey Co., California, USA	010				JN241909	KC262247 (cl 1229)
	Stanislaus Co., California, USA	707					KC262248 (cl 1230)
	San Luis Obispo, Co., California, USA	263					KC262249 (cl 1234)
	San Diego Co., CA	163					KC262250 (cl 1232)
	Reading, UK^*^	205					KC262251 (cl 1231)
							KC262252 (cl 1233)
						JN241897	
						JN241899	
						JN241910	
						JN241913	
					KM491213		
	Reading, UK^*^	205			KM491215		
	San Francisco Co., CA	79G3					
	Reading, UK^*^	205				JN241944 (cl 2541)	
	San Francisco Co., CA	79G3			KM491208		
	Sanpete Co, UT	P199069			MH128480		
	Sanpete Co, UT	P199071			MH128481		
	Sanpete Co, UT	P199072			MH128482		
	Santa Clara Co., CA	245		OQ721997		JN241912	
				OQ721999			
	Van Buren Co., MI		MG821326				
	Costa Rica		KY962665				
*M. natalei*	Spain	-	EU570214				
*M. salasi*	Morocco	-	KP896293				
*M. silvestri*	Netherlands		AF435801				
*M. spartelensis*	OR (garlic mustard)	95F5					MH590652 (cl 3255)
							MH590653 (cl 3256)
		95F6					MH590654 (cl 3260)
							MH590655 (cl 3261)
							MH590656 (cl 3262)
							MH590657 (cl 3263)
*M. trifoliophila*							
*Meloidogyne sp.*							
